# Mentzer Index in breast cancer: Insights into anemia and tumor progression – a narrative review

**DOI:** 10.1097/MD.0000000000045114

**Published:** 2025-10-03

**Authors:** Emmanuel Ifeanyi Obeagu

**Affiliations:** a Department of Biomedical and Laboratory Science, Africa University, Mutare, Zimbabwe.

**Keywords:** anemia, breast cancer, iron deficiency, Mentzer Index, tumor progression

## Abstract

Anemia is a prevalent condition in breast cancer patients, often linked to factors such as chemotherapy, nutritional deficiencies, and tumor-related inflammation. The Mentzer Index, traditionally used to differentiate iron deficiency anemia from thalassemia, has recently gained attention as a tool for diagnosing and managing anemia in oncology. This review explores the application of the Mentzer Index in breast cancer, highlighting its potential role in identifying the underlying causes of anemia and guiding treatment strategies. By assessing the Mentzer Index, clinicians can better understand the nature of anemia in breast cancer patients, which may ultimately improve prognosis and treatment outcomes. The relationship between anemia and tumor progression in breast cancer is complex, with anemia often signaling more aggressive disease and poor prognosis. Iron deficiency, as identified by an elevated Mentzer Index, may exacerbate tumor hypoxia, fostering conditions that promote tumor cell proliferation and metastasis. Conversely, anemia caused by chronic inflammation or cancer-induced suppression of erythropoiesis may further impair immune function and contribute to treatment resistance. The Mentzer Index provides valuable insights into these mechanisms, aiding clinicians in making informed decisions regarding anemia management and its potential impact on tumor dynamics.

## 1. Introduction

Anemia is a common complication among breast cancer patients, impacting their overall health, treatment outcomes, and quality of life.^[1]^ It is associated with various factors such as chemotherapy-induced bone marrow suppression, chronic inflammation, blood loss, and nutritional deficiencies. The presence of anemia often exacerbates symptoms like fatigue and weakness, which can impair a patient’s ability to tolerate cancer treatments and negatively influence their prognosis.^[2]^ While many diagnostic tools exist for assessing anemia, the Mentzer Index (MI) has emerged as a valuable method for distinguishing between different types of anemia, particularly IDA and thalassemia.^[3]^ The MI, a simple formula calculated by dividing the mean corpuscular volume (MCV) by the red blood cell (RBC) count, has traditionally been used to differentiate IDA from thalassemia. A MI >13 typically indicates IDA, while values <13 suggest thalassemia or other types of microcytic anemia. However, recent research has explored the application of the MI beyond its conventional use in general hematology, especially in the oncology setting. In breast cancer, the index can be particularly useful in understanding whether anemia is driven by iron deficiency or if it is a consequence of the cancer itself, possibly due to the tumor’s inflammatory effects or chemotherapy treatment.^[4]^

Iron deficiency, the most common cause of anemia, often requires iron supplementation or intravenous iron therapy. On the other hand, anemia due to chronic disease, including cancer-related anemia, requires addressing the underlying tumor-related inflammation or suppressing the bone marrow’s ability to produce RBCs. The MI can assist clinicians in distinguishing between these causes, leading to more accurate diagnoses and tailored treatment plans that address the specific needs of breast cancer patients.^[4]^ Beyond its utility in diagnosing anemia, the MI may also offer insights into the tumor’s biological behavior.^[5]^ Anemia in cancer patients, particularly iron deficiency anemia (IDA), can lead to hypoxic conditions in tissues, which in turn can promote tumor growth and metastasis.^[6]^ This occurs because tumors require a constant supply of oxygen to proliferate, and when the body experiences anemia, compensatory mechanisms, such as the production of angiogenesis-promoting factors, are activated. Consequently, identifying and treating anemia early through tools like the MI could not only alleviate patient symptoms but also potentially slow tumor progression. Moreover, chronic anemia in breast cancer patients can impair immune function.^[7]^ The inflammatory cytokines involved in cancer-related anemia, such as interleukin-6 (IL-6) and tumor necrosis factor-alpha (TNF-α), contribute to immune system suppression, making it more difficult for the body to fight cancer cells.^[8]^ By differentiating between IDA and anemia due to chronic inflammation, clinicians can gain a better understanding of how anemia may be affecting immune responses, leading to more effective management strategies for improving patient outcomes. In addition to the direct effects on tumor progression, anemia in breast cancer patients also influences their ability to tolerate cancer treatments, particularly chemotherapy.^[9]^ Chemotherapy itself is a common cause of anemia, as it suppresses the bone marrow’s ability to produce RBCs. Anemia may make patients more susceptible to side effects such as fatigue, dizziness, and reduced physical functioning, which can significantly reduce their quality of life. This review aims to explore the role of the MI in breast cancer patients, highlighting its potential in diagnosing and managing anemia, its implications for tumor progression, and its contribution to improving patient care.

### 1.1. Aim

The aim of this review is to explore the role of the MI in the assessment of anemia in breast cancer patients, with a focus on understanding its implications for tumor progression and treatment outcomes.

### 1.2. Rationale

Anemia is a common and debilitating condition in breast cancer patients, often exacerbated by cancer treatments such as chemotherapy, radiation, and surgery.^[6]^ It can significantly affect a patient’s quality of life, treatment adherence, and overall prognosis. The MI, a diagnostic tool traditionally used to distinguish between IDA and thalassemia, has potential value in breast cancer settings for identifying the specific type of anemia and guiding targeted treatment strategies.^[10,11]^ The rationale for this review stems from the need to enhance our understanding of the multifaceted role of anemia in breast cancer, particularly in relation to tumor progression and the effects of various treatments. While the MI has been used primarily in hematology, its application in oncology, specifically breast cancer, remains underexplored. By integrating the MI with existing anemia management strategies, clinicians can more effectively personalize care for breast cancer patients, potentially improving outcomes and minimizing complications. Furthermore, the intersection of anemia and cancer progression is a critical area of research. Anemia in breast cancer may not only result from chemotherapy or nutritional deficiencies but could also be a marker of disease severity or tumor-induced inflammation. This review aims to bridge the gap between the clinical application of the MI and the biological understanding of anemia’s impact on tumor biology. By focusing on this intersection, the review seeks to provide insights into improving therapeutic strategies, enhancing patient care, and optimizing the management of both anemia and breast cancer.

## 2. Review methods

This narrative review was conducted with the objective of exploring the role and clinical implications of the MI in breast cancer, particularly in relation to anemia characterization and tumor progression. To achieve this, a comprehensive and integrative literature search was performed across multiple electronic databases, including PubMed, Google Scholar, Scopus, and ScienceDirect, covering studies published up to March 2025. Keywords and search terms included combinations of: “Mentzer Index,” “breast cancer,” “anemia in cancer,” “iron deficiency,” “thalassemia trait,” “tumor progression,” and “hematological markers in oncology.” Boolean operators such as *AND, OR*, and *NOT* were employed to refine the search. Relevant studies were selected based on their focus on the clinical utility of the MI, anemia subtyping in cancer patients, and hematological changes associated with breast cancer. Articles included in this review encompassed original research papers, clinical studies, retrospective analyses, and review articles that addressed the use of RBC indices in cancer populations. Priority was given to peer-reviewed literature, though gray literature and relevant medical guidelines were also consulted to enrich context and interpretation. Given the narrative nature of this review, a formal quality assessment or meta-analysis was not performed. Instead, findings were synthesized thematically to highlight key trends, implications, and gaps in the current knowledge. The focus remained on providing a broad yet clinically relevant perspective on how the MI may be utilized as a diagnostic and prognostic tool in the care of breast cancer patients with anemia.

## 3. Mentzer Index

The MI is a simple and widely used diagnostic tool in hematology, primarily employed to differentiate between types of microcytic anemia. It is calculated by dividing the MCV by the RBC. The primary clinical application of the MI is to distinguish between IDA and thalassemia, 2 common causes of microcytic anemia. IDA typically results in lower MCV values and a higher RBC count, leading to a MI >13. In contrast, thalassemia, which involves an inherited defect in hemoglobin production, generally results in a lower RBC count and a lower MI, typically below 13. This differentiation is crucial, as it directs clinicians toward appropriate treatment, such as iron supplementation for IDA or genetic counseling for thalassemia.^[12–14]^ The MI has proven to be a useful, cost-effective method for identifying the underlying cause of anemia. By distinguishing between these 2 types of anemia, clinicians can avoid unnecessary iron supplementation in patients with thalassemia and prevent the complications associated with misdiagnosis. Despite its simplicity, the index has limitations, including its inability to account for other causes of microcytic anemia, such as anemia of chronic disease (ACD) or anemia associated with other hematologic conditions.^[15,16]^

## 4. Anemia in breast cancer

Anemia is a frequent and often debilitating complication in breast cancer patients, with significant implications for their overall health, treatment outcomes, and quality of life. It is estimated that approximately one-third of breast cancer patients experience some form of anemia, which can be caused by a variety of factors, including chemotherapy, radiation therapy, nutritional deficiencies, and the cancer itself. Anemia in breast cancer patients is associated with increased fatigue, weakness, and reduced ability to tolerate treatment, which can hinder therapeutic efficacy and affect the patient’s quality of life.^[17]^ Chemotherapy, one of the most common treatments for breast cancer, is a leading cause of anemia. It can suppress the bone marrow’s ability to produce RBCs, leading to a condition known as chemotherapy-induced anemia (CIA). This type of anemia is typically normocytic (normal-sized RBCs) and can be compounded by the toxic effects of chemotherapy on other organs and systems. In addition to chemotherapy, radiation therapy, especially when it targets the pelvis or abdomen, can also damage the bone marrow and exacerbate anemia.^[18]^ IDA is another prevalent cause of anemia in breast cancer patients. Iron deficiency can occur as a result of poor nutrition, blood loss from treatment-related side effects (such as gastrointestinal bleeding), or malabsorption of nutrients due to chemotherapy or other cancer-related factors. In this case, anemia is characterized by microcytic (small-sized) RBCs and low iron stores. ACD, which is often observed in cancer patients, is another contributing factor. ACD is typically normocytic or microcytic and arises from the inflammatory responses associated with cancer, which can suppress erythropoiesis (RBC production) and alter iron metabolism.^[19]^

Treatment strategies may include iron supplementation or intravenous iron therapy for IDA, erythropoiesis-stimulating agents (ESAs) for CIA, or addressing the underlying inflammation and tumor burden in cases of ACD. Differentiating between these various causes of anemia is essential for selecting the most effective therapy and ensuring that patients receive the appropriate support during their cancer treatment.^[20]^ The impact of anemia on breast cancer patients goes beyond just physical symptoms. Anemia has been shown to contribute to poorer prognosis, as it is often linked to more advanced disease and reduced tolerance to treatments like chemotherapy and radiation. Furthermore, anemia can impair immune function, making patients more susceptible to infections and decreasing their overall ability to fight cancer. Therefore, timely diagnosis and effective treatment of anemia in breast cancer patients are critical for optimizing outcomes and improving quality of life.^[21]^ In addition to the well-established causes of anemia, emerging research suggests that tumor-related factors also play a significant role. In breast cancer, the tumor microenvironment can contribute to anemia through a variety of mechanisms. Tumors often release pro-inflammatory cytokines such as IL-6 and TNF-α, which can suppress erythropoiesis and lead to ACD. Additionally, tumors can sequester iron, making it unavailable for RBC production, thereby exacerbating anemia. This interplay between tumor progression and anemia underscores the complexity of managing anemia in breast cancer patients and highlights the need for personalized treatment approaches.^[22]^

## 5. MI and tumor progression

The MI, a simple diagnostic tool primarily used to differentiate between IDA and thalassemia based on the ratio of MCV to RBC, has emerged as a useful parameter in understanding the relationship between anemia and tumor progression, particularly in cancer patients, including those with breast cancer. This index offers insight into the nature of anemia, which is a common and significant concern in oncology, as it can impact both treatment outcomes and overall prognosis. Although originally designed for hematological conditions, the MI can also play a role in distinguishing between anemia due to iron deficiency and anemia associated with cancer-related inflammation, both of which have implications for tumor progression.^[23]^ Anemia in cancer patients, including those with breast cancer, is often linked to a more advanced disease state and poor prognosis. ACD, commonly seen in cancer patients, presents with normocytic or microcytic RBCs and is primarily caused by inflammation and tumor-induced alterations in iron metabolism. In contrast, IDA, characterized by microcytic RBCs and low iron stores, often results from poor nutrition, blood loss, or chemotherapy-induced toxicity. The MI can help differentiate between these 2 types of anemia, providing clinicians with valuable information about the potential underlying causes. This distinction is crucial because anemia related to chronic disease (such as cancer) is typically associated with more severe disease progression, whereas anemia caused by iron deficiency may reflect different management needs.^[24]^

Tumor progression in breast cancer is associated with a variety of factors, including the development of anemia.^[25]^ Inflammatory cytokines released by the tumor and the host immune response can suppress erythropoiesis (RBC production), leading to ACD.^[26]^ This type of anemia can result in a lower MI (indicating a normocytic or microcytic anemia), suggesting that the anemia may be a consequence of cancer-related inflammation rather than nutritional deficiencies. Furthermore, as tumors progress, they can also affect the bone marrow directly through metastasis, reducing its ability to produce RBCs, further contributing to anemia. The relationship between anemia and tumor progression is complex, and the use of the MI provides a useful tool to discern between different forms of anemia that may impact tumor dynamics. For instance, the presence of IDA, indicated by a higher MI, may suggest a more treatable cause, such as dietary insufficiency or blood loss, which could be addressed with iron supplementation. On the other hand, a lower MI, indicative of ACD, may signal a more advanced or aggressive form of cancer, which could require more complex therapeutic interventions, such as ESAs or other strategies aimed at managing the underlying inflammatory processes.

Furthermore, the tumor’s microenvironment plays a critical role in both anemia and tumor progression.^[27]^ Tumors can secrete pro-inflammatory cytokines like IL-6, TNF-α, and interferon-gamma, which suppress the production of erythropoietin, a hormone that stimulates RBC production.^[28]^ This suppression of erythropoiesis contributes to the development of ACD, which, as mentioned, typically presents with a lower MI. This inflammation-driven anemia is often associated with a worse prognosis in cancer patients, including those with breast cancer, as it may indicate more advanced disease and a compromised ability to fight infections or tolerate aggressive treatments like chemotherapy. In terms of tumor progression, the presence of anemia – especially ACD – can serve as a biomarker of the severity of the underlying malignancy.^[29]^ As such, the MI, by helping clinicians understand whether anemia is likely due to iron deficiency or cancer-induced inflammation, could serve as an adjunctive tool for assessing tumor progression. For example, in a patient with a lower MI indicating inflammation-driven anemia, further investigation into the tumor’s cytokine profile and overall burden could provide insights into the aggressiveness of the cancer and help guide treatment decisions. Conversely, if a higher MI points to IDA, addressing the nutritional status or considering iron supplementation might be more appropriate. The MI can also be integrated with other diagnostic and prognostic tools to provide a more comprehensive understanding of tumor progression in breast cancer. For instance, alongside imaging studies, genetic profiling, and inflammatory markers, the MI can offer additional insight into how anemia is influencing or being influenced by tumor growth.

## 6. Clinical implications of the MI in breast cancer

Given that anemia is common among cancer patients, including those with breast cancer, the MI offers valuable insights into the etiology of anemia, which can guide clinical decision-making regarding treatment options and overall management. Differentiating between the types of anemia is crucial in cancer care, as it allows for tailored therapeutic approaches that can improve treatment outcomes, minimize complications, and enhance patient quality of life.^[30]^ In breast cancer patients, anemia can arise from various factors, including chemotherapy, radiation therapy, nutritional deficiencies, and the cancer itself. ACD, which is frequently observed in cancer patients, presents with a normocytic or microcytic pattern and results from tumor-induced inflammation and altered iron metabolism. The MI can help distinguish ACD from IDA, which is typically characterized by microcytic RBCs and low iron stores. A high MI (>13) suggests that iron deficiency is the primary cause, which could be managed with iron supplementation. In contrast, a lower MI (<13) typically indicates anemia due to chronic disease, which requires a different treatment approach focused on managing the underlying cancer or inflammation.^[31]^ The identification of anemia type through the MI can also inform the management of CIA, a common complication in breast cancer patients undergoing treatment. Chemotherapy can suppress bone marrow function, leading to a decrease in RBC production and contributing to normocytic or microcytic anemia. If a patient’s MI is found to be low, it may suggest that chemotherapy-induced bone marrow suppression is the main cause of anemia. In such cases, treatment with ESAs, which promote RBC production, could be considered. On the other hand, if iron deficiency is detected using a higher MI, the focus would shift toward correcting the nutritional deficiency with oral or intravenous iron supplementation.^[32]^

Additionally, the MI can be particularly valuable in the context of anemia in metastatic breast cancer. In patients with advanced or metastatic disease, anemia is often more pronounced and typically associated with the tumor’s cytokine-induced effects, leading to ACD. The MI may help clinicians identify whether the anemia is driven by iron deficiency or cancer-related inflammation. If anemia is determined to be related to cancer-induced inflammation (as suggested by a lower MI), strategies such as addressing the tumor burden, using anti-inflammatory agents, or supporting erythropoiesis with ESAs may be necessary to improve RBC production. Furthermore, understanding the type of anemia can assist in determining the optimal course of treatment to ensure the best possible clinical outcomes in metastatic settings.^[30]^ Another important clinical implication of the MI in breast cancer is its role in optimizing treatment tolerance. Anemia can significantly impact a patient’s ability to tolerate aggressive treatments, including chemotherapy and radiation. Severe anemia can lead to symptoms like fatigue, weakness, and decreased functional capacity, which in turn may necessitate dose reductions, delays in treatment, or cessation of therapy. By accurately diagnosing the underlying cause of anemia through the MI, clinicians can provide targeted interventions, such as iron supplementation or the use of ESAs, to mitigate these symptoms and improve the patient’s overall tolerance to treatment. This not only enhances the patient’s quality of life but also contributes to the success of cancer treatment.^[31]^ In breast cancer patients, especially those with early-stage or localized disease, the management of anemia can directly influence prognosis and treatment efficacy. Anemia can reduce the body’s ability to tolerate radiation and chemotherapy, which can lead to suboptimal treatment regimens and reduced efficacy. By using the MI to differentiate between anemia due to chronic disease and iron deficiency, clinicians can ensure that anemia is managed appropriately, thus minimizing the impact on cancer therapy and improving the chances of successful outcomes.^[32]^The MI is also a useful tool in the management of anemia in the context of supportive care. In cancer care, managing the side effects of treatment and improving the patient’s overall well-being are critical components of care. By helping to identify the type of anemia present, the MI allows clinicians to take a more proactive approach in treating anemia and preventing its exacerbation during cancer treatment. This contributes to a more comprehensive treatment plan that addresses not only the cancer itself but also the side effects of the treatment and the overall health of the patient.

## 7. Potential therapeutic approaches in managing anemia in breast cancer

Anemia is a common complication in breast cancer patients, particularly during and after chemotherapy, and its management is critical to improving overall treatment outcomes, quality of life, and patient prognosis. Various therapeutic approaches can be utilized depending on the underlying cause of anemia, and understanding the specific nature of anemia – whether it is IDA, ACD, or CIA – is essential for tailoring effective treatment strategies. Here are several potential therapeutic approaches for managing anemia in breast cancer patients:

1. Iron supplementation and iron therapy

Iron deficiency is a common cause of anemia in breast cancer patients, particularly when there is blood loss during surgery, chemotherapy, or radiation therapy. In cases where the MI suggests IDA (typically indicated by a higher index), iron supplementation is the first-line therapy. Oral iron supplements, such as ferrous sulfate, are widely used to replenish iron stores in the body and promote RBC production. However, oral iron therapy may cause gastrointestinal side effects and is often less effective in severely anemic patients. For those who cannot tolerate oral iron or require more immediate iron replenishment, intravenous (IV) iron therapy is an effective alternative. IV iron, such as ferric carboxymaltose or iron sucrose, can rapidly increase iron levels and help alleviate anemia in cancer patients. Intravenous iron is particularly useful in patients with malabsorption issues or those undergoing chemotherapy who have a high demand for iron due to the suppression of erythropoiesis (RBC production). Proper iron repletion not only improves RBC count but also helps patients feel less fatigued, enhancing their overall quality of life during cancer treatment.^[33–35]^

2. Erythropoiesis-stimulating agents (ESAs)

ESAs, such as epoetin alfa and darbepoetin alfa, are commonly used to treat anemia associated with chemotherapy. These agents mimic the effects of erythropoietin, a hormone naturally produced by the kidneys that stimulates the production of RBCs in the bone marrow. ESAs are particularly beneficial in patients with CIA, as they help boost RBC production and reduce the need for blood transfusions. While ESAs can effectively address anemia in breast cancer patients, they must be used cautiously due to potential risks, including increased tumor progression and the development of thromboembolic events (blood clots). Clinical guidelines often recommend using ESAs only in patients with CIA who are not candidates for blood transfusion or in those who are not responding to iron supplementation. Close monitoring of hemoglobin levels and careful patient selection are essential to ensure that ESAs are used safely and effectively.^[36–39]^

3. Blood transfusions

In severe cases of anemia, blood transfusions may be necessary, particularly in breast cancer patients who experience significant blood loss or severe anemia during treatment. Transfusions provide an immediate boost to RBC count and hemoglobin levels, improving oxygen delivery to tissues and alleviating symptoms like fatigue and weakness. While blood transfusions can rapidly address the immediate effects of anemia, they do not treat the underlying cause, and repeated transfusions may lead to complications such as iron overload or alloimmunization (development of antibodies against transfused blood products). For breast cancer patients, transfusions are generally reserved for cases where anemia is severe (hemoglobin levels below 7–8 g/dL) or when symptoms significantly impact the patient’s ability to undergo treatment or function daily. Transfusions can be life-saving, but their long-term use should be carefully monitored to avoid complications.^[40,41]^

4. Addressing inflammation and ACD

ACD is frequently observed in cancer patients, including those with breast cancer, and is caused by inflammation and dysregulated iron metabolism. Tumors and the immune response to cancer can produce inflammatory cytokines such as IL-6 and TNF-α, which interfere with iron metabolism and suppress erythropoiesis, leading to anemia. To manage ACD, the focus is on reducing inflammation and improving the body’s ability to utilize iron. While there is no direct pharmacological therapy to reverse ACD, strategies to manage inflammation and support erythropoiesis can help alleviate anemia. Non-steroidal anti-inflammatory drugs (NSAIDs) or targeted therapies that reduce inflammatory cytokine levels, such as IL-6 inhibitors, may have some potential in managing anemia in breast cancer patients. Additionally, the use of ESAs, as discussed earlier, may be beneficial in stimulating RBC production in patients with ACD.^[42,43]^

5. Nutritional support

Nutritional deficiencies, including iron, folate, and vitamin B12 deficiencies, can contribute to anemia in breast cancer patients. Malnutrition, often exacerbated by chemotherapy-induced nausea, vomiting, and loss of appetite, can lead to insufficient intake of essential nutrients required for RBC production. Ensuring adequate nutrition is crucial for preventing and treating anemia in breast cancer patients. Nutritional interventions, including oral supplementation of iron, folate, and vitamin B12, or even specialized diets, can support RBC production and prevent the development of nutritional anemia. In cases where nutritional deficiencies are severe, intravenous vitamin and mineral supplementation may be necessary. Working with a dietitian to monitor and address nutritional deficiencies is an important aspect of managing anemia in breast cancer patients.^[44,45]^

6. Targeted therapies for tumor-induced anemia

Emerging research suggests that targeted therapies that address the mechanisms of tumor-induced anemia could offer new avenues for treatment. For example, therapies aimed at blocking the effects of inflammatory cytokines or modulating the bone marrow’s response to tumor-related factors could help alleviate anemia in cancer patients. Additionally, therapies that enhance iron availability, such as hepcidin antagonists, could be beneficial in treating ACD. Targeted therapies could also involve enhancing the body’s natural production of erythropoietin or improving iron absorption and utilization at the cellular level. Ongoing clinical trials are investigating novel agents that could more directly address the underlying causes of anemia in breast cancer, offering hope for more personalized and effective treatments in the future.^[46,47]^

7. Combination approaches

In many cases, a combination of therapeutic strategies may be required to manage anemia effectively in breast cancer patients. For instance, patients with both iron deficiency and chronic disease-related anemia may benefit from a combination of iron supplementation and ESAs. Similarly, blood transfusions may be used in the short term to manage severe anemia, while longer-term treatments such as ESAs or iron therapy address the underlying causes. Comprehensive treatment plans that combine pharmacological interventions with nutritional support, management of inflammation, and symptom relief are essential for optimizing anemia care in breast cancer patients. The goal is to provide a holistic approach that addresses the anemia and the cancer itself, improving the patient’s ability to tolerate treatment and ultimately improving their prognosis.^[48,49]^

## 8. The role of the MI in the management of anemia in breast cancer and tumor progression

**Figure 1.**
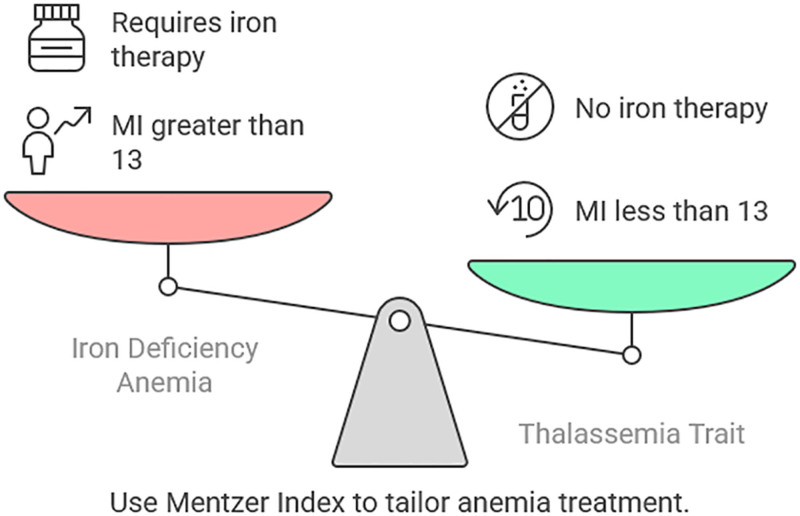
Use of Mentzer Index to tailor anemia treatment in breast cancer.

Anemia is a common and clinically significant comorbidity in patients with breast cancer, affecting treatment outcomes, quality of life, and overall survival. The causes of anemia in this population are multifactorial – ranging from nutritional deficiencies and chronic inflammation to bone marrow suppression due to chemotherapy or tumor infiltration. Differentiating the underlying type of anemia is crucial, as each requires a tailored therapeutic approach. It is within this diagnostic framework that the Mentzer Index (MI) emerges as a simple yet powerful tool.^[12,50]^ The MI, calculated by dividing the MCV by the RBC count, is traditionally used to distinguish IDA from thalassemia trait. In the context of breast cancer, this distinction becomes particularly relevant. IDA, the most common subtype in cancer patients, is often exacerbated by chronic disease, inflammation, or gastrointestinal blood loss – factors that are prevalent among breast cancer patients, especially those undergoing chemotherapy. A MI >13 generally indicates IDA, suggesting the need for iron supplementation or further work-up for blood loss. Conversely, an index <13 suggests thalassemia trait, which may not require iron therapy and could prevent inappropriate treatment.^[32,51,52]^ In managing breast cancer, early identification and correction of anemia using tools like the MI can significantly improve patient outcomes. Anemic patients often experience reduced tolerance to chemotherapy, increased fatigue, and delayed recovery. Correctly categorizing the anemia ensures that supportive care measures – such as iron therapy, ESAs, or blood transfusions – are accurately applied, thus preventing treatment interruptions and enhancing therapeutic efficacy.^[31,53]^ Beyond its diagnostic utility, emerging insights suggest that the MI may also indirectly reflect tumor dynamics. As tumors grow and progress, they often induce systemic inflammation and nutritional deficiencies, leading to microcytic or normocytic anemia. Changes in red cell indices, including MCV and RBC count, can be early hematological indicators of such systemic alterations. In some observational studies, breast cancer patients with a high MI were found to have more advanced disease stages, possibly due to prolonged inflammation and poor nutritional status.^[30,32,54,55]^ This highlights the potential role of the MI not just as a diagnostic tool, but as a surrogate marker for disease severity or progression (Fig. 1).

## 9. MI in breast cancer: insights into anemia and tumor progression

Distinguishing the underlying type of anemia is crucial, as it informs both the clinical management plan and prognostic expectations. Among the hematological tools available, the MI, though traditionally applied in distinguishing IDA from thalassemia, is gaining attention in oncology as a potentially valuable and accessible parameter for evaluating anemia in breast cancer patients. The MI is calculated by dividing the MCV by the RBC count. Values >13 typically indicate IDA, while values below 13 suggest a thalassemia trait. However, its relevance extends further when interpreted in the context of malignancy. In breast cancer, patients often experience ACD, which is primarily mediated by systemic inflammation. This inflammation, driven by tumor-associated cytokines such as interleukin-6 (IL-6), leads to elevated levels of hepcidin – a liver-derived peptide hormone that inhibits iron absorption and release. Consequently, even with adequate iron stores, functional iron becomes unavailable for erythropoiesis, resulting in anemia that mimics iron deficiency but does not respond to iron supplementation.^[32,51–53]^ Herein lies the potential utility of the MI. In breast cancer patients, a high MI may indeed reflect true iron deficiency, often arising from poor dietary intake, malabsorption, or chronic blood loss. On the other hand, a moderately elevated MI that does not correlate with low ferritin levels or positive response to iron therapy may point towards inflammation-induced functional iron deficiency. This distinction is clinically relevant. Treating pure IDA with oral or intravenous iron can restore hemoglobin levels and improve quality of life. However, iron supplementation in the context of cancer-related inflammation may be ineffective or, in some cases, detrimental if excess iron contributes to oxidative stress or supports tumor cell proliferation.^[30,31]^

**Figure 2.**
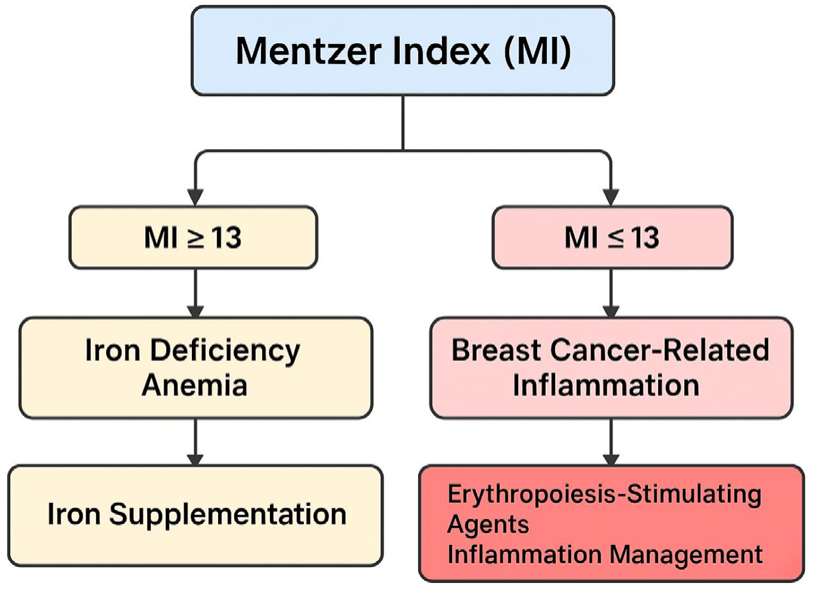
A diagram which shows the role of MI in distinguishing between anemia due to iron deficiency and anemia associated with cancer-related inflammation, and guiding treatment and management options. MI = Mentzer Index.

Furthermore, the MI can serve as a practical screening tool in resource-limited settings where more specific assays such as serum ferritin, transferrin saturation, or hepcidin assays may be unavailable. A persistently high MI, particularly when MCV is reduced and RBC count is not elevated, may warrant further iron studies. Conversely, a normal or low MI with normocytic or microcytic indices could indicate a non-iron-deficiency etiology, steering the clinician toward evaluating chronic disease anemia or the impact of cytotoxic therapies on marrow function.^[54]^ The implications for treatment are considerable. In patients with a confirmed iron deficiency (MI > 13, low ferritin, high TIBC), iron therapy can be initiated, either orally or intravenously depending on severity and tolerance. In contrast, patients with inflammatory anemia (e.g., MI > 13 but with high ferritin and low serum iron) might benefit more from anti-inflammatory approaches, ESAs, or careful monitoring during cancer treatment. Moreover, understanding the type of anemia can help optimize chemotherapy dosing schedules, as severe anemia can delay treatment or exacerbate fatigue and reduce treatment adherence.^[32]^ From a prognostic perspective, anemia in breast cancer is associated with tumor hypoxia, which can drive angiogenesis, metastatic potential, and resistance to therapy through the activation of hypoxia-inducible factors [Fig. 2]. Thus, early identification and correction of anemia, guided in part by tools like the MI, may have implications beyond symptomatic relief, potentially impacting long-term outcomes.^[55]^

## 10. Recommendations

1. **Early screening and monitoring of anemia**

Regular screening for anemia, particularly during the early stages of breast cancer treatment, is crucial for timely intervention. Clinicians should use diagnostic tools like the MI to distinguish between different types of anemia and tailor treatment strategies accordingly. Routine monitoring of hemoglobin levels, iron status, and erythropoietin levels should be conducted throughout the treatment process, especially in patients undergoing chemotherapy or radiation therapy.

2. **Personalized therapeutic approaches**

Given the variety of causes behind anemia in breast cancer patients, personalized treatment plans should be developed based on the specific type of anemia diagnosed. For example, IDA can be managed effectively with oral or intravenous iron supplementation, while CIA may benefit from the use of ESAs.

3. **Combining therapies for optimal outcomes**

A combination of treatments may be necessary to manage anemia in breast cancer patients effectively. This could include iron supplementation alongside ESAs for those with both iron deficiency and ACD, or the use of blood transfusions in severe cases where immediate relief is required. By adopting a comprehensive, multi-pronged approach, healthcare providers can address both the anemia and its underlying causes, minimizing symptoms and improving patients’ quality of life.

4. **Targeted therapies for tumor-induced anemia**

As research into tumor-induced anemia progresses, targeted therapies designed to address the molecular mechanisms of anemia could provide new treatment avenues. Developing therapies that reduce inflammation, enhance iron metabolism, or stimulate erythropoiesis directly within the tumor microenvironment could lead to more effective management of anemia in breast cancer patients. Continued research into the role of cytokines and other factors contributing to anemia is essential to creating more precise, targeted treatment options.

5. **Collaboration between oncologists, hematologists, and dietitians**

Multidisciplinary collaboration is key to effectively managing anemia in breast cancer patients. Oncologists, hematologists, and dietitians should work together to monitor the progression of anemia, adjust treatment plans as needed, and address nutritional deficiencies. Ensuring that patients receive adequate nutrition, including necessary vitamins and minerals, can help prevent or manage anemia while supporting overall health during cancer treatment. Collaborative care can also help mitigate the side effects of anemia treatments, enhancing patient adherence and outcomes.

6. **Patient education and support**

Educating patients about the signs and symptoms of anemia, as well as the importance of adhering to prescribed therapies, is essential for effective management. Breast cancer patients should be informed about the potential side effects of anemia treatments, including the risk of iron overload from excessive supplementation or complications from blood transfusions. Emotional and psychological support should also be provided to help patients cope with the physical and emotional challenges posed by anemia and cancer treatment.

7. **Long-term follow-up care**

Anemia in breast cancer patients may persist beyond the completion of active treatment. Therefore, long-term follow-up care to monitor for the recurrence of anemia and its impact on overall health is recommended. Continued vigilance can help identify any lingering anemia issues or complications, such as iron overload from multiple transfusions or side effects from long-term use of ESAs, allowing for timely interventions to improve patient outcomes.

## 11. Conclusion

This narrative review set out to evaluate the role and clinical implications of the MI in breast cancer, particularly as it relates to anemia characterization and potential insights into tumor behavior. The MI, traditionally employed to distinguish between IDA and thalassemia, emerges as a valuable yet underutilized tool in the oncology setting. In breast cancer patients, the MI can aid in identifying the underlying etiology of anemia – whether related to nutritional deficits, chronic inflammation, or marrow involvement – thereby guiding more targeted and effective management strategies. Furthermore, the MI may serve as a simple, accessible hematological marker that reflects systemic changes associated with tumor progression. Its association with anemia severity, inflammatory status, and possibly even breast cancer subtypes, suggests that it holds promise not only for diagnostic clarification but also for informing prognosis and monitoring treatment response.

## Author contributions

**Conceptualization:** Emmanuel Ifeanyi Obeagu.

**Methodology:** Emmanuel Ifeanyi Obeagu.

**Resources:** Emmanuel Ifeanyi Obeagu.

**Supervision:** Emmanuel Ifeanyi Obeagu.

**Validation:** Emmanuel Ifeanyi Obeagu.

**Visualization:** Emmanuel Ifeanyi Obeagu.

**Writing – original draft:** Emmanuel Ifeanyi Obeagu.

**Writing – review & editing:** Emmanuel Ifeanyi Obeagu.
